# Macrophages in trigeminal ganglion contribute to ectopic mechanical hypersensitivity following inferior alveolar nerve injury in rats

**DOI:** 10.1186/s12974-017-1022-3

**Published:** 2017-12-16

**Authors:** Dulguun Batbold, Masamichi Shinoda, Kuniya Honda, Akihiko Furukawa, Momoko Koizumi, Ryuta Akasaka, Satoshi Yamaguchi, Koichi Iwata

**Affiliations:** 1 Department of Oral and Maxillofacial Surgery, Graduate School of Medical and Dental Sciences, Tokyo Medical and Dental University, 1-5-45 Yushima, Bunkyo-ku, Tokyo, 113-8549 Japan; 2 Department of Physiology, School of Dentistry, Nihon University, 1-8-13 Kandasurugadai, Chiyoda-ku, Tokyo, 101-8310 Japan; 3 Department of Clinical Medicine, School of Dentistry, Nihon University, 1-8-13 Kandasurugadai, Chiyoda-ku, Tokyo, 101-8310 Japan; 4 Department of Dentistry, Jikei University School of Medicine, 3-25-8 Nishi-Shimbashi, Minato-ku, Tokyo, 105-8461 Japan

**Keywords:** Trigeminal ganglion, Tumor necrosis factor alpha, Macrophage, Ectopic orofacial pain, Inferior alveolar nerve transection, Mechanical allodynia

## Abstract

**Background:**

Accidental mandibular nerve injury may occur during tooth extraction or implant procedures, causing ectopic orofacial pain. The exact mechanisms underlying ectopic orofacial pain following mandibular nerve injury is still unknown. Here, we investigated the role of macrophages and tumor necrosis factor alpha (TNFα) in the trigeminal ganglion (TG) in ectopic orofacial pain following inferior alveolar nerve transection (IANX).

**Methods:**

IANX was performed and the mechanical head-withdrawal threshold (MHWT) in the whisker pad skin ipsilateral to IANX was measured for 15 days. Expression of Iba1 in the TG was examined on day 3 after IANX, and the MHWT in the whisker pad skin ipsilateral to IANX was measured following successive intra-ganglion administration of the macrophage depletion agent liposomal clodronate Clophosome-A (LCCA). TNFα expression in the TG and the MHWT in the whisker pad skin ipsilateral to IANX following successive intra-ganglion administration of the TNFα blocker etanercept were measured on day 3 after IANX, and tumor necrosis factor receptor-1 (TNFR1) immunoreactive (IR) cells in the TG were analyzed immunohistochemically on day 3.

**Results:**

The MHWT in the whisker pad skin was significantly decreased for 15 days, and the number of Iba1-IR cells was significantly increased in the TG on day 3 after IANX. Successive intra-ganglion administration of the macrophage depletion agent LCCA significantly reduced the increased number of Iba1-IR cells in the TG and reversed the IANX-induced decrease in MHWT in the whisker pad skin. TNFα expression was increased in the TG on day 3 after IANX and was reduced following successive intra-ganglion administration of the TNFα inhibitor etanercept. The decreased MHWT was also recovered by etanercept administration, and TNFR1-IR cells in the TG were increased on day 3 following IANX.

**Conclusions:**

These findings suggest that signaling cascades resulting from the production of TNFα by infiltrated macrophages in the TG contributes to the development of ectopic mechanical allodynia in whisker pad skin following IANX.

## Background

There are several causes of orofacial neuropathic pain, including accidental nerve injury or compression of the trigeminal nerve during dental treatments such as dental implant surgery and tooth extractions or facial trauma [1–3]. The orofacial pain may occasionally spread to intact orofacial regions, and clinicians struggle to alleviate the symptoms of ectopic orofacial pain. However, the detailed mechanisms of ectopic neuropathic pain are not fully understood.

Previous studies have indicated that pathological changes in the intra-trigeminal ganglion (TG) signaling mechanisms following trigeminal nerve injury are involved in the development of ectopic orofacial neuropathic pain. For example, nitric oxide released from the injured TG neurons and satellite glial activation via connexin 43 signaling modulates the excitability of intact TG neurons following inferior alveolar nerve injury (IANX) [4, 5]. Recently, immune cells that are infiltrated following peripheral nerve injury have been shown to play an important and diverse role in pathological neuronal changes. Sciatic nerve transection induces macrophage proliferation around axotomized dorsal root ganglion neurons and contributes to the regeneration of transected afferent nerve fibers [6–8]. Furthermore, sciatic nerve injury-induced macrophage invasion into the injured site and injury-induced mechanical and thermal hypersensitivity was abolished by macrophage depletion [9, 10]. The infiltration of activated macrophages into the dorsal root ganglion results in the development of behavioral hypersensitivity in rats which induced chemotherapy-induced peripheral neuropathy [11].

Tumor necrosis factor alpha (TNFα) is well known as a proinflammatory cytokine involved in the development and maintenance of neuropathic pain. Increased TNFα was observed in the injured region in a model of sciatic nerve injury-induced mechanical and thermal hyperalgesia, and inhibition of TNFα synthesis abolished the hyperalgesia [12]. Activated macrophages are known to produce and release TNFα [13]. These results suggest that TNFα production by infiltrating macrophages may be involved in pain hypersensitivity following peripheral nerve injury.

Therefore, we hypothesized that the proliferation of activated macrophages in the TG contributes to the development of orofacial ectopic neuropathic pain following inferior alveolar nerve transection (IANX). In this study, we examined the relation between mechanical allodynia following IANX with the infiltration of macrophages into the TG and elucidated their functional roles in the development of ectopic orofacial neuropathic pain.

## Methods

### Animals

We used male Sprague-Dawley rats (*n* = 101, Japan SLC, Shizuoka, Japan) weighing 170–260 g. All rats were housed individually in transparent polycarbonate cages (length 48 cm; width 26.5 cm; height 21 cm) with paper shavings as bedding. The room temperature was controlled (23 °C) with a 12 h light/dark cycle (lights on at 7:00, off at 19:00), and animals were allowed ad libitum access to food and water. All experiments were performed according to the National Institutes of Health Guide for the Care and Use of Laboratory Animals and the guidelines of the International Association for the Study of Pain [14]. This study was approved by the Animal Experimentation Committee at Nihon University (AP16D010).

### Inferior alveolar nerve transection

IANX was performed as described previously under deep anesthesia with intraperitoneal (i.p.) butorphanol (2.5 mg/kg, Meiji Seika Pharma, Tokyo, Japan), medetomidine (0.375 mg/kg, Zenoaq, Fukushima, Japan), and midazolam (2.0 mg/kg, Sandoz, Tokyo, Japan) [15]. Briefly, a small incision was made in the skin of the left cheek, and the masseter muscle was dissected to expose the mandibular bone surface. The surface of the mandible was scraped using a low-speed dental drill bur to expose the inferior alveolar nerve. The exposed inferior alveolar nerve was gently pulled, transected, and replaced into the mandibular canal. In the control group, the skin incision, muscle dissection, and bone scraping without IANX were performed (sham operation). The dissected muscle and skin incision were sutured with 6-0 silk.

### Measuring mechanical sensitivity of the whisker pad skin

Rats were trained daily for 5 to 7 days to protrude their snout from a cage that had a small fenestration and to withdraw their snout freely following application of mechanical stimuli to the whisker pad skin by von Frey filaments (Touch-Test Sensory Evaluator, North Coast Medical, Morgan Hill, CA) as described previously [4]. After stabilizing the mechanical head-withdrawal threshold (MHWT), IANX or sham operation was performed and followed by behavioral testing under the same conditions every other day for 15 days. The MHWT was determined as the lowest intensity that evoked head-withdrawal responses to five stimuli (duration 1 s). The behavioral testing was conducted under blinded conditions.

### Intra-TG administration of Clophosome-A and etanercept

Under deep anesthesia with i.p. butorphanol (2.5 mg/kg), medetomidine (0.375 mg/kg), and midazolam (2.0 mg/kg), a head of rat was placed in a stereotaxic instrument for guidance [4, 16]. The skull was exposed and a small hole (1 mm in diameter) was drilled above the location of the TG (2.8 mm anterior from the posterior fontanelle, 2.7 mm lateral to the sagittal suture) ipsilateral to the location of IANX or sham operation. A guide cannula was extended in the TG (9 mm below the skull surface) and was anchored to the skull bone with dental cement and three stainless-steel screws. To confirm the correct positioning of the tip of the cannula, a trocar was inserted into the cannula as an electrode and multi-unit activities induced by mechanical stimulation of the whisker pad skin were conformed. After implantation of the cannula, rats were allowed to recover for 5 days before performing the IANX or sham operation. Under light anesthesia with 2% isoflurane in oxygen, liposomal clodronate Clophosome-A (LCCA, 6 μl, 7 mg/mL, product code: F70101CA, FormuMax Scientific, Sunnyvale, CA, USA) or plain control liposomes for LCCA (Cont-LCCA, 6 μl, 20 mM, product code: F70101-A, FormuMax Scientific) were administered into the TG through the guide cannula prior to operation and on days 1 to 4 following IANX or sham operation. LCCA has been shown to be effective in successfully depleting proliferated and resident macrophages [17]. Etanercept (1 μg; Pfizer, New York, NY, USA) was dissolved in physiological saline or vehicle alone (physiological saline) and administered (6 μl) into the TG through the guide cannula under light anesthesia with 2% isoflurane in oxygen prior to operation and on days 1 to 4 following IANX or sham operation. The LCCA and etanercept administration were performed immediately after the measurement of the MHWT of whisker pad skin. The MHWT of the whisker pad skin was measured before operation and every other day for 5 days after IANX or sham operation.

Moreover, rats were allowed to recover after implantation of the cannula for 5 days. Under light anesthesia with 2% isoflurane in oxygen, 2 μl of 1,1′-dioctadecyl-3,3,3′,3-tetramethylindocarbocyanine methanesulfonate (DiI, Thermo Fisher Scientific, Waltham, MA) was administered into the TG through the guide cannula. On day 3 after the administration, rats were deeply anesthetized with the above-described solution and transcardially perfused with saline followed by a fixative containing 4% paraformaldehyde in 0.1 M phosphate buffer (PB; pH 7.4). After the perfusion, TGs were removed and immersed in a fixative containing 4% paraformaldehyde for more than 4 h at 4 °C then transferred to 0.01 M phosphate-buffered saline (PBS) for 12 h for cryoprotection. TG from naive rat was also processed in a similar manner. Next, the tissues were embedded in Tissue-Tek (Sakura Finetechnical, Tokyo, Japan) and were cut in 12-μm-thick sections in a horizontal plane along the long axis.

### Immunohistochemistry

Under deep anesthesia with i.p. butorphanol (2.5 mg/kg), medetomidine (0.375 mg/kg), and midazolam (2.0 mg/kg), a retrograde labeling tracer, FluoroGold (7 μl dissolved in saline; FG; 4% hydroxystilbamidine; Fluorochrome, Denver, CO, USA), was injected into the skin of the left whisker pad ipsilateral to IANX using a 30-gauge needle before performing the IANX or sham operation. On day 3, rats were deeply anesthetized with the above-described solution and transcardially perfused with saline followed by a fixative containing 4% paraformaldehyde in 0.1 M phosphate buffer (PB; pH 7.4). After the perfusion, TGs were removed and immersed in the same fixative for more than 4 h at 4 °C then transferred to 0.01 M PBS containing 20% sucrose for 12 h for cryoprotection. Next, the tissues were embedded in Tissue-Tek and stored at − 20 °C until cryosectioning. Then, the ganglion tissues were cut in 12-μm-thick sections in a horizontal plane along the long axis. Every 10th section (five sections per rat) was thaw-mounted on a MAS-GP slide glass (Matsunami, Osaka, Japan) and dried overnight at room temperature. The five sections were used for immunohistological analysis. After rinsing with 0.01 M PBS three times for 5 min each, sections were incubated in mouse anti-Iba1 polyclonal antibody (1:200; Abcam, Cambridge, MA, USA) and rabbit anti-TNFα monoclonal antibody (1:200; Abcam) or rabbit anti-glial fibrillary acidic protein (GFAP) polyclonal antibody (1:500; cat.No. ab7260, Abcam) or rabbit anti-F4/80 monoclonal antibody (1:500; cat.No. ab111101, Abcam), or anti-tumor necrosis factor receptor-1 (TNFR1) monoclonal antibody (1:50; Santa Cruz, Santa Cruz, CA, USA), diluted in 0.01 M PBS containing 4% normal donkey serum (Merck, Darmstadt, Germany) in 0.3% Triton X-100 (Merck) overnight at 4 °C. After rinsing with 0.01 M PBS, the sections were incubated in Alexa Fluor 568 anti-rabbit IgG (1:200; Thermo Fisher Scientific) and/or Alexa Fluor 488 anti-mouse IgG (1:200; Thermo Fisher Scientific) in 0.01 M PBS for 2 h at room temperature. After rinsing with 0.01 M PBS, sections were coverslipped in PermaFluor (Thermo Fisher Scientific). The sections were observed using a BZ-9000 system (Keyence, Osaka, Japan). Iba1-IR and TNFα-IR cells in the TGs of the ophthalmic-maxillary division (V1-V2) and mandibular division (V3) were analyzed. The mean relative area (density) of Iba1-IR or TNFα-IR cells were calculated by using a computer-assisted imaging analysis system (ImageJ, NIH, Bethesda, MD, USA). The mean relative area (density) of F4/80- and Iba1-IR area was calculated by using a computer-assisted imaging analysis system (BZ-X analyzer, Keyence, Osaka, Japan). All immunohistochemical measurements were conducted under blinded conditions, performed by observers who were unaware of which treatment the animals received. Using the same conditions, no specific labeling was observed in the absence of primary antibody. Measurement of the area occupied by the immuno-products was made in a region selected identically from V1/V2 and V3 in the TG.

### Western blotting

On day 3 following IANX or sham operation, the rats were perfused through the aorta with physiological saline under deep anesthesia as described above. Immediately, the TG was removed and homogenized in ice-cold lysis buffer (137 mM NaCl; 20 mM Tris-HCL, pH 8.0; 1% NP40; 10% glycerol; 1 mM phenylmethylsulfonyl fluoride; 10 μg/ml aprotinin; 1 g/ml leupeptin; 0.05 mM sodium vanadate). The homogenate was centrifuged at 15,000 rpm for 10 min at 4 °C, and the protein concentration of the resulting supernatants was determined using a protein assay kit (Bio-Rad, Hercules, CA, USA). The supernatants were heat-denatured in Laemmli sample buffer solution (Bio-Rad), and 30 μg of each protein sample was subjected to SDS-PAGE using a 10% acrylamide gel. The samples were transferred to a polyvinylidene difluoride membrane (Trans-Blot Turbo Transfer Pack, Bio-Rad) using Trans-Blot Turbo (Bio-Rad). The membrane was rinsed with Tris-buffered saline mixed with 0.1% Tween 20 (TBST) and incubated in 3% bovine serum albumin (BSA; Bovogen, Essendon, Australia). The membrane was then incubated with rabbit anti-TNFα polyclonal antibody (1:200; diluted in TBST and 3% BSA, Abcam) or rabbit anti-Iba1 monoclonal antibody (1:1000; diluted in TBST and 3% BSA, cat.No. ab178847, Abcam) overnight at 4 °C. Next, it was incubated with horseradish peroxidase-conjugated donkey anti-rabbit antibody (Cell Signaling, Danvers, MA, USA) for 2 h at room temperature. Antibody binding was detected using Western Lightning ELC Pro (PerkinElmer, Waltham, MA, USA). Band intensity was analyzed using a ChemiDoc MP system (Bio-Rad) and normalized to β-actin on blots re-probed with the anti-β-actin antibody (1:200; Santa Cruz) after removing bound protein using a stripping reagent (Thermo Scientific).

### Statistical analysis

Data were expressed as mean ± standard error. Statistical analyses were performed by Student’s *t* test, one-way analysis of variance (ANOVA) with Tukey’s multiple-comparison test, and two-way ANOVA with repeated measures followed by Bonferroni’s multiple-comparison test where appropriate. A value of *p* < 0.05 was defined as statistically significant.

## Results

### Mechanical allodynia and Iba1 expression in TG following IANX

**Fig. 1:**
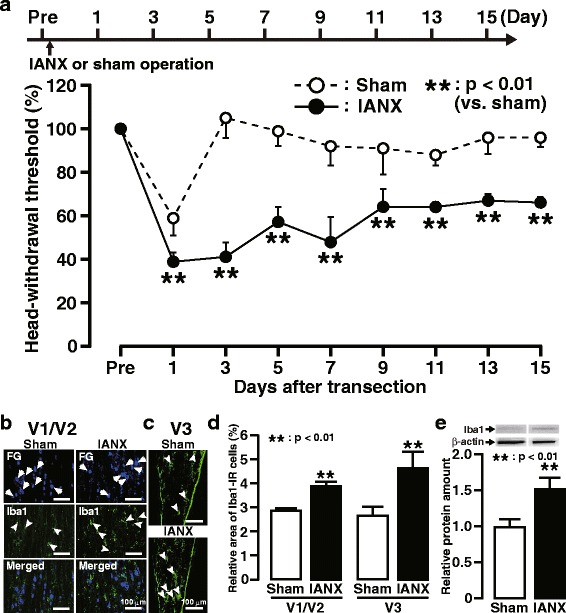
Mechanical allodynia and Iba1 expression in the TG following IANX. **a** The timing of IANX or sham operation (upper) and the changes in MHWT of the ipsilateral whisker pad skin for 15 days following IANX or sham operation (lower). The MHWT was normalized to the pre-IANX MHWT (100%). Error bars indicate SEM. **p < 0.01 vs. sham (*n* = 6 in each; two-way ANOVA with repeated measures followed by Bonferroni’s multiple-comparison test). Iba1-IR cells in V1/V2 (**b**) and V3 (**c**) in the ipsilateral TG on day 3 following IANX or sham operation. Arrows indicate FG-labeled neurons. Arrowheads indicate Iba1-IR cells. **d** The relative area of Iba1-IR cells in V1/V2 and V3 in TG on day 3 following IANX and sham operation. Error bars indicate SEM. **p < 0.01 vs. sham. (*n* = 5 in each, Student’s *t* test). **e** The relative amount of Iba1 protein in TG on day 3 following IANX or sham operation. Error bars indicate SEM. *p < 0.05 vs. sham (*n* = 10 in each, Student’s *t* test)

The MHWT of the whisker pad skin ipsilateral to IANX was significantly decreased on day 1 following IANX, and this decrease persisted until day 15 when compared with sham-operated animals (Fig. 1a). On day 3 following IANX, Iba1-IR cells were observed in V1/V2 FG-labeled neurons, as well as in V3, in which FG-labeled neurons were absent (Fig. 1b, c). The relative area of Iba1-IR cells in V1/V2 and V3 in TG ipsilateral to IANX was significantly increased on day 3 following IANX compared to that of the sham-operated group (Fig. 1d). The Iba1 protein level was increased in TG ipsilateral to IANX on day 3 following IANX compared to sham-operated animals (Fig. 1e).

### Effect of intra-TG LCCA administration on mechanical allodynia and Iba1 expression following IANX

**Fig. 2:**
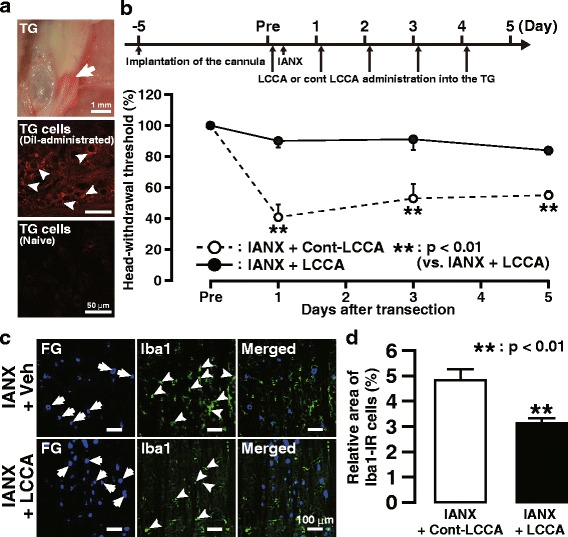
Effect of intra-TG LCCA administration on mechanical allodynia and Iba1 expression following IANX. **a** Photograph (upper) and photomicrograph (middle) of TG on day 3 following DiI administration into the TG through the guide cannula and photomicrograph (lower) of TG in naive rat. Arrows indicate DiI labeling. **b** The timing of the cannula implantation, LCCA, or Cont-LCCA administration into the TG, IANX, or sham operation (upper) and the changes in MHWT of the ipsilateral whisker pad skin following daily intra-TG LCCA or Cont-LCCA administration following IANX (lower). The MHWT was normalized to the pre-IANX MHWT (100%). Arrows indicate the timing of intra-TG LCCA or Cont-LCCA administration. Error bars indicate SEM. **p < 0.01 vs. sham (*n* = 6 in each; two-way ANOVA with repeated measures followed by Bonferroni’s multiple-comparison test). **c** Iba1-IR cells and FG-labeled TG neurons in V1/V2 on day 3 following intra-TG LCCA or Cont-LCCA administration in IANX rats. Arrows indicate FG-labeled neurons. Arrowheads indicate Iba1-IR cells. **d** The relative area of Iba1-IR cells in V1/V2 in TG on day 3 following intra-TG LCCA or Cont-LCCA administration in IANX rats. Error bars indicate SEM. **p < 0.01 vs. sham. (*n* = 5 in each, Student’s *t* test)

Primarily, it was confirmed that drug administration into the TG through the guide cannula was accurate (Fig. 2a). Daily administration of LCCA into the TG prevented the development and progress of mechanical allodynia in the whisker pad skin ipsilateral to IANX from days 1 to 5 following IANX (Fig. 2b). On day 3 after IANX, Iba1-IR cells in V1/V2, in which FG-labeled neurons were abundant, were observed in the LCCA- and Cont-LCCA-treated groups (Fig. 2c). Moreover, the relative area of Iba1-IR cells in the LCCA-treated group was significantly lower compared to the group treated with Cont-LCCA (Fig. 2d). Before intra-TG LCCA and Cont-LCCA administration, it was confirmed that the guide cannula implantation did not change the mechanical sensitivity (data not shown).

### TNFα expression in TG following IANX

**Fig. 3:**
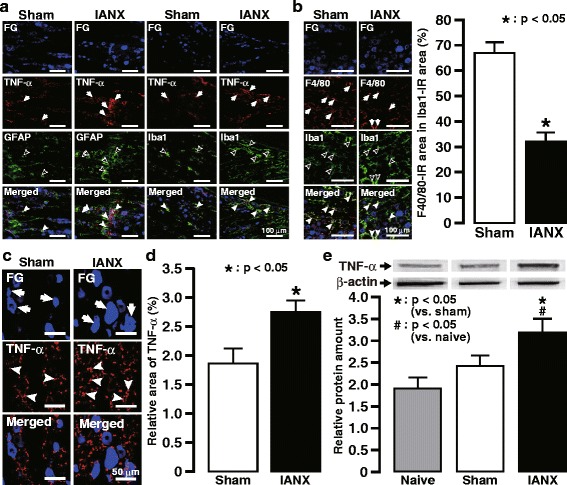
TNFα expression in TG following IANX. **a** TNFα expression in the Iba1-IR cells or the GFAP-IR cells in ipsilateral TG on day 3 following IANX or sham operation. Arrows indicate TNFα-IR cells, open arrowheads indicate GFAP-IR cells or Iba1-IR cells, and close arrowheads indicate TNFα- and GFAP-IR cells or TNFα- and Iba1-IR cells. **b** F4/80 expression in the Iba1-IR cells in ipsilateral TG on day 3 following IANX or sham operation (left). Arrows indicate F4/80 expression in Iba1-IR cells, open arrowheads indicate Iba1-IR cells, and close arrowheads indicate F4/80- and Iba1-IR cells. The relative area of F4/80 immuno-products in Iba1-IR cells in the TG on day 3 following IANX or sham operation (right). Error bars indicate SEM. ***p < 0.001 vs. sham. (*n* = 5 in each, Student’s *t* test). **c** TNFα expression in ipsilateral TG on day 3 following IANX or sham operation. Arrows indicate FG-labeled neurons. Arrowheads indicate TNFα immuno-products. **d** The relative area of TNFα immuno-products in the TG on day 3 following IANX or sham operation. Error bars indicate SEM. *p < 0.05 vs. sham (*n* = 5 in each, Student’s *t* test). **e** The relative amount of TNFα protein in TG in naive animals or on day 3 following IANX or sham operation. Error bars indicate SEM. *p < 0.05 vs. sham. #*p* < 0.05 vs. naive (*n* = 5 in each, one-way ANOVA with Tukey’s multiple-comparison test)

**Fig. 4:**
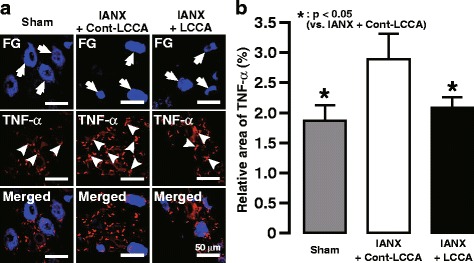
Changes in TNFα expression in TG following LCCA administration following IANX. **a** TNFα expressed in V1/V2 in the TG on day 3 following intra-TG LCCA or Cont-LCCA administration in IANX rats or in sham-operated rats. Arrows indicate FG-labeled neurons. Arrowheads indicate TNFα immuno-products. **b** Changes in the relative area of TNF-α immuno-products in the TG on day 3 following intra-TG LCCA or Cont-LCCA administration in IANX rats or in sham-operated rats. Error bars indicate SEM. *p < 0.05 vs. Cont-LCCA. (*n* = 5 in each, one-way ANOVA with Tukey’s multiple-comparison test)

TNFα was expressed in activated Iba1-IR cells presented with morphological characteristics such as the bigger cell bodies and thicker ramifications and in activated GFAP-IR cells in V1/V2 on day 3 in the IANX group rather than sham group (Fig. 3a). F4/80 immunoreactivity was observed in the Iba1-IR cells in V1/V2 on day 3 in IANX or sham groups, and the relative F4/80-IR area in the Iba1-IR area was decreased in V1/V2 ipsilateral to IANX compared to that of the sham-operated group (Fig. 3b). The relative area of TNFα immuno-products increased in V1/V2 ipsilateral to IANX compared to that of the sham-operated group (Fig. 3c, d). The TNFα protein level was also increased in TG ipsilateral to IANX on day 3 following IANX compared to naive and sham-operated animals (Fig. 3e). Daily LCCA administration significantly reduced the relative area of TNFα immuno-products in V1/V2 in TG on day 3 following IANX compared to the Cont-LCCA-treated IANX group or sham group (Fig. 4a, b).

### Effect of intra-TG etanercept administration in TG on mechanical allodynia following IANX

**Fig. 5:**
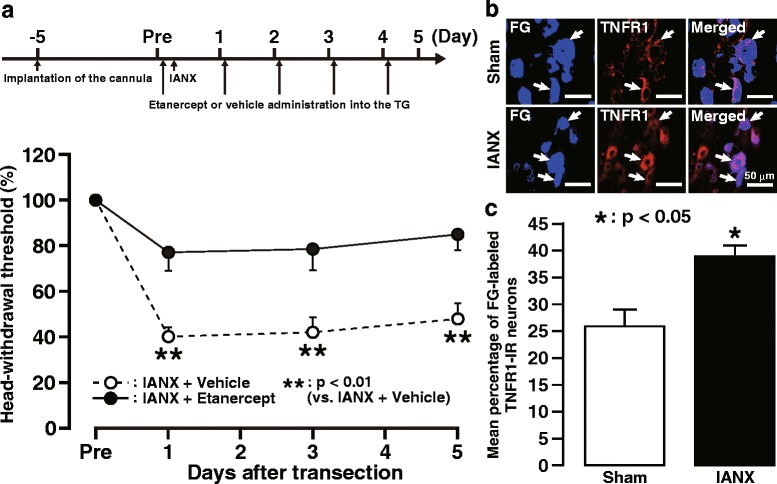
Effect of intra-TG etanercept administration on mechanical allodynia following IANX. **a** The timing of the cannula implantation, etanercept, or vehicle administration into the TG, IANX, or sham operation (upper) and changes in MHWT of the ipsilateral whisker pad skin by daily etanercept administration into TG following IANX (lower). The MHWT was normalized to the pre-IANX MHWT (100%). Arrows indicate the timing of intra-TG etanercept or vehicle administration. Error bars indicate SEM. **p < 0.01 vs. IANX + vehicle (*n* = 6 in each; two-way ANOVA with repeated measures followed by Bonferroni’s multiple-comparison test). **b** TNFR1 immunoreactivity in ipsilateral TG on day 3 following IANX or in sham-operated rats. Arrows indicate FG-labeled TNFR1-IR neurons. **c** Changes in the mean percentage of FG-labeled TNFR1-IR neurons on day 3 following IANX. Error bars indicate SEM. *p < 0.05 vs. sham (*n* = 5 in each, Student’s *t* test)

Daily administration of etanercept into the ipsilateral TG prevented mechanical hypersensitivity in whisker pad skin ipsilateral to IANX from days 1 to 5 (Fig. 5a). Immunostaining was performed to examine TNFR1 expression in the TG on day 3 following IANX. TNFR1 expression was observed in FG-labeled neurons, and the mean percentage of FG-labeled TNFR1-IR neurons in TG ipsilateral to IANX was significantly increased compared to the sham-operated group (Fig. 5b).

## Discussion

Traumatic injury to the inferior alveolar nerve, which can occur during extraction of an impacted mandibular third molar or mandibular plastic surgery, occasionally causes ectopic pain hypersensitivity in widespread orofacial areas such as the forehead and maxillary regions [1]. In this study, IANX produced long-lasting mechanical allodynia in ipsilateral whisker pad skin, as reported in our previous studies [4, 15, 18]. The long-lasting mechanical allodynia observed in the IANX model mimics ectopic pain hypersensitivity in patients with a history of traumatic injury to the inferior alveolar nerve, indicating that this model may be useful for research into the mechanisms of ectopic orofacial neuropathic pain.

Iba1 expression is known to be widely used as a specific macrophage marker [19]. IANX induced extensive macrophage infiltration in not only V3 but also in V1/V2 and increased Iba1 expression in the ipsilateral TG. Moreover, the infiltrated macrophages showed the bigger cell bodies and thicker ramifications, which indicates their activation [20]. Previous studies have shown that nerve injury increases resident and proliferated macrophages in the sensory ganglion [21–23]. The chemokine C-C motif ligand 2 (CCL2), also referred to as monocyte chemoattractant protein 1, is released from injured neurons and activates the C-C chemokine receptor type 2 (CCR2) in macrophages, which induces macrophage activation and proliferation in the dorsal root ganglion (DRG) following peripheral nerve injury [8, 24]. Moreover, CCL2 hyper-expression and macrophage infiltration in the DRG, induced by peripheral nerve injury, is reduced in Toll-like receptor 2 (TLR2) knock-out mice [25]. Based on these results, we hypothesize that the extensive infiltration of activated macrophages in the TG ipsilateral to IANX observed here was induced by CCL2 released from injured neurons via TLR2 signaling. F4/80 expression is known to be used as tissue-resident macrophage marker [26, 27]. The relative F4/80-IR area in the Iba1-IR area in TG was decreased following IANX in this study, suggesting that the proliferation of the activated macrophages in TG ipsilateral to IANX is mainly caused by exogenous macrophage infiltration into the TG.

The proliferation of activated macrophages was observed in the TG ipsilateral to IANX, and these activated macrophages expressed TNFα. IANX significantly increased TNFα expression in the ipsilateral TG, and macrophage depletion in the TG significantly suppressed the increase in TNFα expression. Macrophages are believed to be the major source of TNFα in the sensory ganglion following peripheral nerve injury [28, 29]. Intracellular signaling cascades such as p38 mitogen-activated protein kinase (MAPK) and extracellular signal-regulated kinase (ERK) cascades result in macrophage activation and TNFα release [30]. The expression of substance P (SP), which is a member of the tachykinin family and a major mediator of neuroimmunomodulation, was found to be enhanced in the sensory ganglion neurons, and SP was released in the early stages after peripheral nerve injury [31]. Neurokinin 1 receptor which is activated by SP is present in macrophage, SP modulates diverse functions of macrophages, and SP signaling activates the ERK 1/2 and p38 MAPK cascades [32–34]. Moreover, the activation of the p38 MAPK cascade and the ensuing TNFα production are induced in macrophages infiltrating into the nerve-injured site [35]. Previous studies have reported that TNFα levels in DRG increased following peripheral nerve injury [36, 37]. Taken together, these results suggest that SP released from TG neurons is transmitted to macrophages, resulting in macrophage activation and TNFα release from activated macrophages via the ERK 1/2 and p38 MAPK signaling cascades. Furthermore, GFAP immunoreactivity which is a marker of the activated satellite glial cells was observed in the TG ipsilateral to IANX, and these activated satellite glial cells also expressed TNFα. Our previous report also indicates that activation of satellite glial cells in the TG was induced after IANX [4], suggesting that the activated satellite glial cells might be another source of TNFα in the TG after IANX. Further studies are needed.

In this study, TNFR1 expression in TG neurons innervating the whisker pad skin and the increase of TNFα in V1/V2 in the TG were observed following IANX. The continuous suppression of TNFα signaling and macrophage depletion in the TG prevented the development and progress of mechanical allodynia in the whisker pad skin ipsilateral to IANX. It was reported that intra-DRG administration of exogenous TNFα depolarizes DRG neurons and increases their neuronal excitability [38]. TNFα signaling in uninjured DRG neurons mediates the activation of voltage-gated sodium channels (Nav) in a dose-dependent manner by upregulating current densities of tetrodotoxin (TTX)-sensitive and TTX-resistant Nav 1.3 and Nav 1.8 in the neurons via p38 MAPK cascade following peripheral nerve injury [36, 39, 40]. TNFα also increases membrane potassium ion conductance in a non-voltage-gated fashion, resulting in the enhancement of neuronal excitability [41, 42]. We have previously reported that satellite glial cells are activated throughout the TG via gap junctions following IANX [4]. Adenosine triphosphate was released from sensory neurons following peripheral nerve injury, and TNFα was released from activated satellite glial cells through P2X_7_ signaling [43, 44]. Based on the existing reports, this study suggests that TNFα released from proliferated macrophages or satellite glial cells binds to TNFR1 in TG neurons, and TNFα-TNFR1 signaling might contribute to the enhancement of TG neuronal excitability, resulting in mechanical allodynia in the whisker pad skin following IANX, whereas IANX significantly increased TNFα expression in not only the activated macrophages but also in the satellite cells other than the activated macrophages in TG. Some reports indicated that peripheral nerve injury induced TNFα hyper-expression in the satellite cells in DRG, suggesting that TNFα released from the satellite cells following IANX also modulates mechanical sensitivity in the whisker pad skin which occurs as a result of TG neuronal excitability [45, 46].

TNFR1 expression in TG neurons innervating the whisker pad skin was significantly increased following IANX, and neutralizing TNFR1 attenuated thermal and mechanical allodynia following peripheral nerve injury [47]. Moreover, TNFR1 knockout mice do not develop mechanical allodynia induced by peripheral nerve injury [36]. Though these reports indicate that TNFR1 signaling plays a pivotal role in the development of mechanical allodynia and contributor to the enhancement of TNFR1 expression in TG neurons innervating the whisker pad skin following IANX, the detailed mechanism remains to be elucidated.

## Conclusions

The signaling cascades induced by TNFα released from infiltrated macrophages in the TG contribute to the development of orofacial neuropathic ectopic pain following IANX. Therefore, TNFα signaling in the TG should be considered as one of the primary targets in the modulation and treatment of ectopic orofacial pain attributed to peripheral trigeminal nerve injury.

## Abbreviations

ANOVA: Analysis of variance
BSA: Bovine serum albumin
CCL2: Chemokine C-C motif ligand 2
CCR2: C-C chemokine receptor type 2
Cont-LCCA: Plain control liposomes for LCCA
DRG: Dorsal root ganglion
ERK: Extracellular signal-regulated kinase
FG: FluoroGold
i.p.: Intraperitoneal
IANX: Inferior alveolar nerve transection
IR: Immunoreactive
LCCA: Liposomal clodronate Clophosome-A
MAPK: Mitogen-activated protein kinase
MHWT: Mechanical head-withdrawal threshold
Nav: Voltage-gated sodium channels
PB: Phosphate buffer
PBS: Phosphate-buffered saline
SP: Substance P
TBST: Tris-buffered saline mixed with 0.1% Tween 20
TG: Trigeminal ganglion
TLR2: Toll-like receptor 2
TNFR1: Tumor necrosis factor receptor-1
TNFα: Tumor necrosis factor alpha
TTX: Tetrodotoxin
V1-V2: Ophthalmic-maxillary division
V3: Mandibular division
